# Genetic variants associated with ventricular arrhythmias during ajmaline test in Brugada syndrome

**DOI:** 10.1093/europace/euaf162

**Published:** 2025-08-05

**Authors:** Charles Audiat, Luigi Pannone, Antonio Sorgente, Pasquale Vergara, Domenico Giovanni Della Rocca, Ivan Eltsov, Gudrun Pappaert, Ingrid Overeinder, Gezim Bala, Alexandre Almorad, Erwin Ströker, Mark La Meir, Ali Gharaviri, Elke De Schutter, Jelle Vlaeminck, Christine Helsen, Sophie Uyttebroeck, Sonia Van Dooren, Laura Pölsler, Philippe Giron, Pedro Brugada, Andrea Sarkozy, Gian Battista Chierchia, Juan Sieira, Carlo de Asmundis

**Affiliations:** Heart Rhythm Management Centre, Postgraduate Program in Cardiac Electrophysiology and Pacing, Universitair Ziekenhuis Brussel—Vrije Universiteit Brussel, European Reference Networks Guard-Heart, Laarbeeklaan 101, Brussels 1090, Belgium; Department of Cardiology, Centre Hospitalier Universitaire Saint-Pierre, Université Libre de Bruxelles, Brussels, Belgium; Heart Rhythm Management Centre, Postgraduate Program in Cardiac Electrophysiology and Pacing, Universitair Ziekenhuis Brussel—Vrije Universiteit Brussel, European Reference Networks Guard-Heart, Laarbeeklaan 101, Brussels 1090, Belgium; Heart Rhythm Management Centre, Postgraduate Program in Cardiac Electrophysiology and Pacing, Universitair Ziekenhuis Brussel—Vrije Universiteit Brussel, European Reference Networks Guard-Heart, Laarbeeklaan 101, Brussels 1090, Belgium; Heart Rhythm Management Centre, Postgraduate Program in Cardiac Electrophysiology and Pacing, Universitair Ziekenhuis Brussel—Vrije Universiteit Brussel, European Reference Networks Guard-Heart, Laarbeeklaan 101, Brussels 1090, Belgium; Heart Rhythm Management Centre, Postgraduate Program in Cardiac Electrophysiology and Pacing, Universitair Ziekenhuis Brussel—Vrije Universiteit Brussel, European Reference Networks Guard-Heart, Laarbeeklaan 101, Brussels 1090, Belgium; Cardiac Surgery Department, Universitair Ziekenhuis Brussel—Vrije Universiteit Brussel, Brussels, Belgium; Heart Rhythm Management Centre, Postgraduate Program in Cardiac Electrophysiology and Pacing, Universitair Ziekenhuis Brussel—Vrije Universiteit Brussel, European Reference Networks Guard-Heart, Laarbeeklaan 101, Brussels 1090, Belgium; Heart Rhythm Management Centre, Postgraduate Program in Cardiac Electrophysiology and Pacing, Universitair Ziekenhuis Brussel—Vrije Universiteit Brussel, European Reference Networks Guard-Heart, Laarbeeklaan 101, Brussels 1090, Belgium; Heart Rhythm Management Centre, Postgraduate Program in Cardiac Electrophysiology and Pacing, Universitair Ziekenhuis Brussel—Vrije Universiteit Brussel, European Reference Networks Guard-Heart, Laarbeeklaan 101, Brussels 1090, Belgium; Heart Rhythm Management Centre, Postgraduate Program in Cardiac Electrophysiology and Pacing, Universitair Ziekenhuis Brussel—Vrije Universiteit Brussel, European Reference Networks Guard-Heart, Laarbeeklaan 101, Brussels 1090, Belgium; Heart Rhythm Management Centre, Postgraduate Program in Cardiac Electrophysiology and Pacing, Universitair Ziekenhuis Brussel—Vrije Universiteit Brussel, European Reference Networks Guard-Heart, Laarbeeklaan 101, Brussels 1090, Belgium; Cardiac Surgery Department, Universitair Ziekenhuis Brussel—Vrije Universiteit Brussel, Brussels, Belgium; Heart Rhythm Management Centre, Postgraduate Program in Cardiac Electrophysiology and Pacing, Universitair Ziekenhuis Brussel—Vrije Universiteit Brussel, European Reference Networks Guard-Heart, Laarbeeklaan 101, Brussels 1090, Belgium; Clinical Sciences, Research Group Reproduction and Genetics, Centre for Medical Genetics, Vrije Universiteit Brussel (VUB), Universitair Ziekenhuis Brussel (UZ Brussel), Brussels, Belgium; Clinical Sciences, Research Group Reproduction and Genetics, Centre for Medical Genetics, Vrije Universiteit Brussel (VUB), Universitair Ziekenhuis Brussel (UZ Brussel), Brussels, Belgium; Clinical Sciences, Research Group Reproduction and Genetics, Centre for Medical Genetics, Vrije Universiteit Brussel (VUB), Universitair Ziekenhuis Brussel (UZ Brussel), Brussels, Belgium; Clinical Sciences, Research Group Reproduction and Genetics, Centre for Medical Genetics, Vrije Universiteit Brussel (VUB), Universitair Ziekenhuis Brussel (UZ Brussel), Brussels, Belgium; Clinical Sciences, Research Group Reproduction and Genetics, Centre for Medical Genetics, Vrije Universiteit Brussel (VUB), Universitair Ziekenhuis Brussel (UZ Brussel), Brussels, Belgium; Clinical Sciences, Research Group Reproduction and Genetics, Vrije Universiteit Brussel (VUB), Universitair Ziekenhuis Brussel (UZ Brussel), Brussels Interuniversity Genomics High Throughput Core (BRIGHTcore), Brussels, Belgium; Clinical Sciences, Research Group Reproduction and Genetics, Centre for Medical Genetics, Vrije Universiteit Brussel (VUB), Universitair Ziekenhuis Brussel (UZ Brussel), Brussels, Belgium; Clinical Sciences, Research Group Reproduction and Genetics, Centre for Medical Genetics, Vrije Universiteit Brussel (VUB), Universitair Ziekenhuis Brussel (UZ Brussel), Brussels, Belgium; Heart Rhythm Management Centre, Postgraduate Program in Cardiac Electrophysiology and Pacing, Universitair Ziekenhuis Brussel—Vrije Universiteit Brussel, European Reference Networks Guard-Heart, Laarbeeklaan 101, Brussels 1090, Belgium; Heart Rhythm Management Centre, Postgraduate Program in Cardiac Electrophysiology and Pacing, Universitair Ziekenhuis Brussel—Vrije Universiteit Brussel, European Reference Networks Guard-Heart, Laarbeeklaan 101, Brussels 1090, Belgium; Heart Rhythm Management Centre, Postgraduate Program in Cardiac Electrophysiology and Pacing, Universitair Ziekenhuis Brussel—Vrije Universiteit Brussel, European Reference Networks Guard-Heart, Laarbeeklaan 101, Brussels 1090, Belgium; Heart Rhythm Management Centre, Postgraduate Program in Cardiac Electrophysiology and Pacing, Universitair Ziekenhuis Brussel—Vrije Universiteit Brussel, European Reference Networks Guard-Heart, Laarbeeklaan 101, Brussels 1090, Belgium; Heart Rhythm Management Centre, Postgraduate Program in Cardiac Electrophysiology and Pacing, Universitair Ziekenhuis Brussel—Vrije Universiteit Brussel, European Reference Networks Guard-Heart, Laarbeeklaan 101, Brussels 1090, Belgium

**Keywords:** Brugada syndrome, Ajmaline test, Ventricular arrhythmias, Sudden cardiac death, Genetics

## Abstract

**Graphical Abstract:**
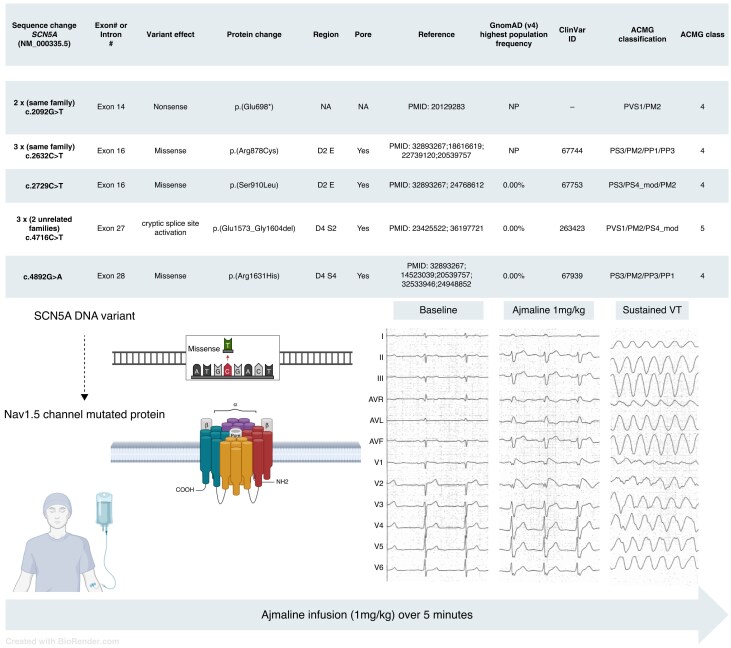
Pathogenic/Likely Pathogenic variants identified in *SCN5A* and associated with ajmaline-induced ventricular fibrillation following ACMG guidelines

Pathogenic/Likely Pathogenic variants identified in *SCN5A* and associated with ajmaline-induced ventricular fibrillation following ACMG guidelines

Ventricular arrhythmias (VA) occur in approximately 2–18% of patients with Brugada syndrome (BrS) undergoing sodium channel blocker (SCB) testing.^1,2^ While these arrhythmias have been linked to the *SCN5A* gene,^1^ the impact of specific gene variants and their long-term clinical outcomes remains poorly understood.^3,4,5^

All consecutive patients with BrS were prospectively enrolled in the UZ Brussel monocentric BrS registry (NCT05283759). Inclusion criteria were (i) BrS diagnosis, (ii) genetic analysis for *SCN5A*, and (iii) recent re-classification of gene variants following current ACMG guidelines.^6^ Ajmaline testing (AJT) was performed in all patients with a standard protocol of 1 mg/kg over 5 min. Ajmaline administration was halted upon the appearance of a type I electrocardiogram (ECG) pattern or if QRS prolongation exceeded 30%. Patients with a history of spontaneous type I ECG were excluded. The AJT-induced VA was defined as ventricular fibrillation (VF) or sustained ventricular tachycardia (VT). The primary endpoint was VA occurrence at follow-up, defined as sudden cardiac death (SCD), VT, VF, or appropriate implantable cardioverter defibrillator (ICD) intervention. The data underlying this article will be shared on reasonable request to the corresponding author.

A total of 465 patients were included (mean age at diagnosis 39.3 ± 16.7 years, 52.3% males), with 18 patients (3.9%) experiencing AJT-induced VA and 447 patients (96.1%) without AJT-induced VA. One external DC shock was required to terminate the arrhythmia in all patients with AJT-induced VA, except in one patient requiring three shocks and two patients requiring multiple shocks. One patient eventually underwent a veno-arterial extracorporeal membrane oxygenator (ECMO) placement to restore sinus rhythm. An ICD was implanted in all patients with VA during AJT. Patients experiencing AJT-induced VA were younger (27.4 years± 19.0 vs. 39.4 years ± 16.7, *P* = 0.03) and had a higher incidence of SCD in the family [9 patients (50.0%) vs. 61 patients (13.6%), *P* < 0.001], a personal history of aborted SCD [5 patients (27.8%) vs. 14 patients (3.1%), *P* < 0.001], a longer PQ interval (192.0 ms ± 32.3 vs. 162.2 ms ± 30.6, *P* < 0.001), and a longer QRS interval (110.0 ms ± 11.2 vs. 98.8 ms ± 17.8, *P* = 0.016). Patients with AJT-induced VA more frequently had a pathogenic/likely pathogenic (P/LP) variant in *SCN5A* compared to the rest of the cohort [10 patients (55.6%) vs. 74 patients (16.6%), *P* < 0.001] (*Figure 1*). A total of 5 different P/LP *SCN5A* (NM_000335.5) variants were identified in 10 AJT-induced VA patients, 4/5 in the pore region. Two related patients (aunt and nephew) were carriers of the c.2092G>T variant. Among the other two family members carrying the c.2092G>T variant, one patient did not experience VA (proband), and in one patient, AJT was not performed due to concern for VA. Three patients had the c.2632C>T variant and were all members of the same family. Another three patients had the c.4716C>T variant; two patients were related (mother and daughter), and one was unrelated (patient with refractory VF and ECMO placement). Each of the other two patients was a carrier of one of the remaining variants.

**Figure 1:**
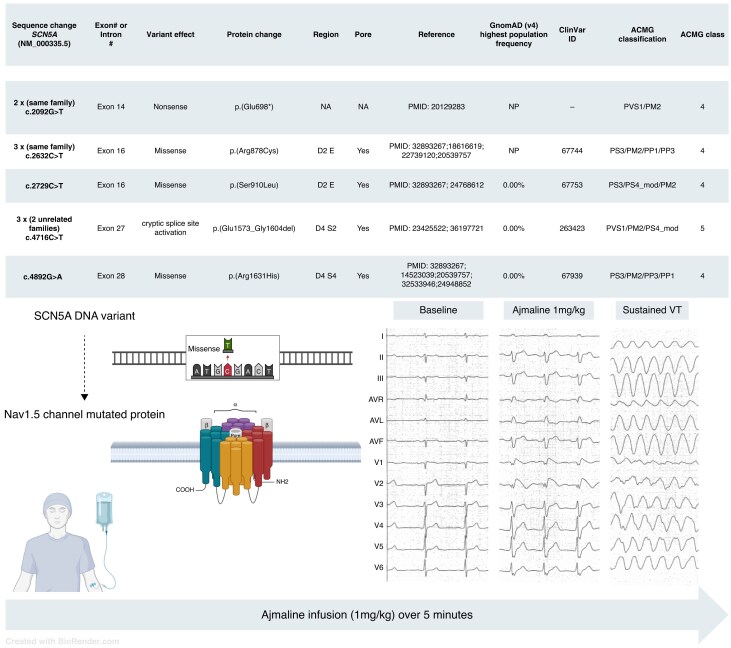
*SCN5A* (NM_000335.5) variants associated with ajmaline-induced ventricular arrhythmias.

At a median follow-up of 109.2 months, AJT-induced VA patients experienced more VA compared with the remaining cohort [8 patients (44.4%) vs. 30 patients (6.7%), *P* < 0.001]. All the events occurred in patients with ICD. A total of 14 patients (16.7%) with a P/LP *SCN5A* variant experienced a VA. At Cox multivariate analysis, independent predictors of VA occurrence were as follows: history of syncope [hazard ratio (HR) = 2.75, 95% confidence interval (CI) 1.35–5.62, *P* = 0.006], history of aborted SCD (HR = 14.12, 95% CI 6.16–32.4, *P* < 0.001), VA inducibility at EPS (HR = 5.96, 95% CI 2.59–13.72, *P* < 0.001), and AJT-induced VA (HR = 5.47, 95% CI 2.24–13.37, *P* < 0.001).

The incidence of AJT-induced VA in the current study is consistent with our previous data^1^ and, with a meta-analysis, reporting a weighted average of 2.4%.^2^ Although the incidence is relatively low, ECMO was required in one patient, highlighting the necessity for advanced life support facilities available during AJT. The incidence of VA at long-term follow-up is consistent with previous reports.^5,7,8^

In two previous studies, *SCN5A* variants were found more frequently in BrS patients with AJT-induced VA.^1^ However, the role of specific gene variants has remained unclear due to small sample sizes. The current study represents the most extensive report on *SCN5A* gene variants associated with AJT-induced VA. In particular, three variants were found in multiple patients experiencing VA, suggesting a possible role. Given the very high risk of VA (up to 12% in this study), the recent consensus statement on the role of pharmacological provocation testing^9^ recommended against performing an AJT in carriers of a P/LP *SCN5A* variant. The variants reported in the current study should be considered as high risk.

The prognosis of patients who developed VA during SCB testing has been evaluated by Chinushi *et al*.^10^ and Conte *et al*.^1^ with no significant difference in prognosis between patients who did and did not develop VA. However, the mean follow-up was relatively short (45 and 29 months, respectively). In the current study, with a median follow-up of 109 months from a registry spanning over 30 years, AJT-induced VA patients had a worse prognosis compared with patients without VA during AJT. Consequently, an ICD should be recommended for BrS patients experiencing VA during SCB testing.

In conclusion, AJT-induced VA in BrS can be associated with specific *SCN5A* gene variants and is associated with a higher long-term arrhythmic risk. Ajmaline testing should be avoided in patients with high-risk variants. The current study does not demonstrate a role for AJT in predicting arrhythmic risk during follow-up in lower-risk individuals (e.g. family members). Therefore, its use in this context cannot be recommended.
